# Graphene based scaffolds effects on stem cells commitment

**DOI:** 10.1186/s12967-014-0296-9

**Published:** 2014-10-25

**Authors:** Eriberto Bressan, Letizia Ferroni, Chiara Gardin, Luca Sbricoli, Luca Gobbato, Francesco Saverio Ludovichetti, Ilaria Tocco, Amedeo Carraro, Adriano Piattelli, Barbara Zavan

**Affiliations:** Department of Neurosciences, University of Padova, via Giustiniani 2, 35131 Padova, Italy; Department of Biomedical Sciences, University of Padova, via Giuseppe Colombo 3, 35131 Padova, Italy; Institute of Plastic Surgery, University Hospital of Padova, via Giustiniani 2, 35131 Padova, Italy; General Surgery and Liver Transplant Unit Department of General Surgery and Odontoiatrics, University Hospital of Verona, P.le A. Stefani 1, 37126 Verona, Italy; Department of Medical, Oral and Biotechnological Sciences, University of Chieti-Pescara, Via dei Vestini 1, 66100 Chieti, Italy

**Keywords:** Graphene, Tissue engineering, Stem cells, Oosteogenic differentiation, Neuronal differentiation, Adipogenic differentiation

## Abstract

Graphene is a flat monolayer of carbon atoms, arranged in a two-dimensional hexagonal structure, with extraordinary electrical, thermal, and physical properties. Moreover, the molecular structure of graphene can be chemically modified with molecules of interest to promote the development of high-performance devices. Although carbon derivatives have been extensively employed in industry and electronics, their use in regenerative medicine is still in an early phase. Study prove that graphene is highly biocompatible, has low toxicity and a large dosage loading capacity. This review describes the ability of graphene and its related materials to induce stem cells differentiation into osteogenic, neuronal, and adipogenic lineages.

## Introduction

An outburst of research on regenerative medicine has recently emerged to develop nanostructured materials as smart interfaces to be used for cellular studies and regenerative medicine. Tissue regeneration is a demanding field in terms of development of biomaterials since it requires a variety of fabrication scales, ranging from signal transduction levels to macroscopic tissue recapitulation. Current nanoscale research is focusing primarily on new materials that might be manufactured at high production volume and thus be associated with significant human application. The final goal is a better understanding of the complexity that entails the native extracellular matrix (ECM) through the creation of in vitro models, leading to engineered scaffolds specifically designed to modulate cells differentiation. Many authors have demonstrated that cell shape, morphology, attachment, proliferation, and migration can be controlled by cell-material interactions [1–4]. The relevant properties of biomaterials in the modulation of cell behavior are not limited to substrate rigidity, topography or roughness; the density and distribution of adhesive ligands, and the chemistry and the substrate elasticity may also induce the up-regulation of neurogenic, myogenic, and osteogenic markers in human mesenchymal stem cells (MSCs) [5].

In the search for brand new factors which may have an influence on cell behavior, much attention has been recently given to environmental components [6,7], and particularly on graphene [8]. Graphene is a flat monolayer of carbon atoms, arranged in a two-dimensional hexagonal structure [9]. The carbon atom has a valence of four, which determines the number of possible covalent bonds between carbon atoms within a molecule. Carbon allotropes differ according to the types of linkages that form between the carbon atoms to create macromolecular structures, for example rolled graphene sheets represent carbon nanotubes (CNT), with extraordinary electrical, thermal, and physical properties. Also, the molecular structure of graphene can be chemically modified, enabling the attachment of different molecules of interest; this feature promotes the development of high-performance devices.

Although carbon derivatives have been extensively employed in industry and electronics, their use in regenerative medicine is still in an early phase [10]. The strategies developed so far to apply carbon-based nanomaterials to tissue engineering and cell differentiation are suspension of nanomaterials into cell culture media or coating nanomaterials for in vitro stem cell culture. The second strategy is widely accepted to modulate stem cell behaviors since nanomaterial-coated substrates are able to provide a unique physical framework, comparable to natural ECM, for stem cell [11,12]. Graphene can improve the performance of a broad range of devices and can be used as an “any-shape” biocompatible single-atomic thick scaffold to enhance stem cells differentiation thanks to its unique physical, chemical and mechanical characteristics [13]. Specifically, graphene is highly biocompatible, has low toxicity and a large dosage loading capacity, making it a potential efficient carrier for therapeutic proteins [14].

**Figure 1:**
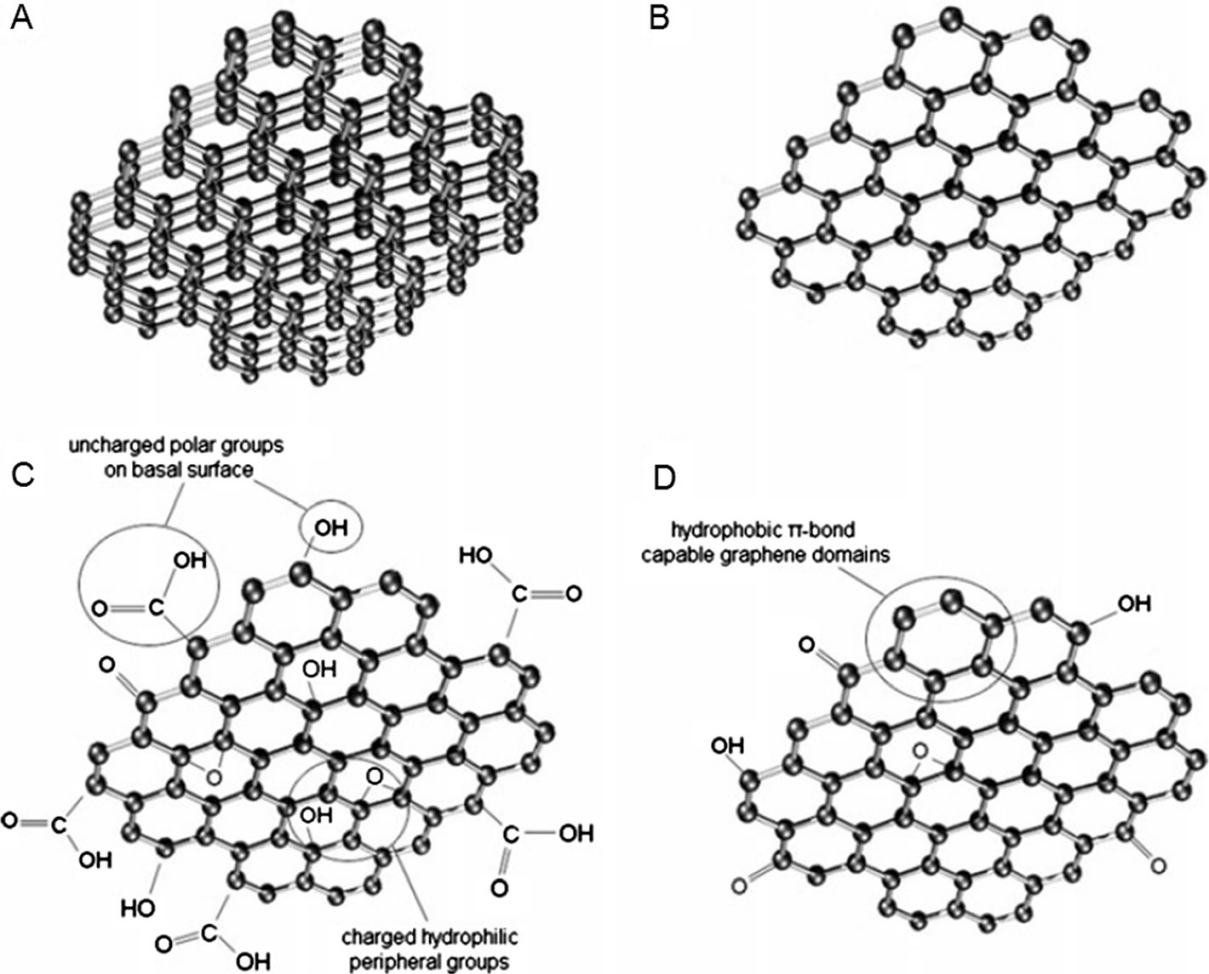
**Representation of some members of the graphene-family nanomaterials.** Few-layered graphene **(A)**, graphene nanosheet **(B)**, graphene oxide (GO) **(C)**, and reduced graphene oxide (rGO) **(D)** [15].

The exceptional properties of graphene and its potential different applications have led to the development of composite materials that include few-layer-graphene (FLG), ultrathin graphite, graphene oxide (GO), reduced graphene oxide (rGO), and graphene nanosheets (GNS), comprising a broad set of “graphene-family nanomaterials” (GFNs) (Figure 1) [15–17].

Some of them, like GO substrates, have been already demonstrated to stimulate human MSCs to differentiate into adypocites [13,18], to induce the differentiation of neural stem cells (NSCs) into neurons in three dimensional (3D) porous structure [19] and of induced pluripotent stem cells into the endodermal lineage [9]. It was also reported that graphene scaffolds may be considered as great substrates to induce bone formation from human MSCs [3]. We further highlight how the properties of graphene are being exploited for stem cell differentiation in tissue engineering, comprehensively surveying recent experimental works featuring graphene and graphene derivatives (Table 1).

**Table 1:** **Commitment of stem cells on different graphene substrates**

**Test substrate**	**Control substrate**	**Cell type**	**Commitment**	**Methods**	**Result**	**Ref.**
Glass slides, PET, PDMS, Si/SiO_2_ coated with graphene sheets	Glass slides, PET, PDMS, Si/SiO_2_	Human MSCs	Osteogenic	Cell Viability assay; Immunofluorescence staining; Alizarin Red staining	In presence of an osteogenic medium, graphene coating helps human MSCs to differentiate to osteogenic phenotype.	[3]
SiO_2_ substrate coated with graphene films	SiO2 substrate	Human MSCs; Human osteoblast-like cell line	Osteogenic	Raman spectroscopy; Immunofluorescence staining	Graphene induces osteoblast cell proliferation: on graphene coated substrates the initial number of cells almost duplicates, while on the SiO_2_ substrate it reaches a factor of 1,5.	[21]
Ti coated with GO sheets	Ti	Human BMMSCs	Osteogenic	Immunofluorescence staining; Implantation in mouse calvarial defects	The osteogenetic differentiation of human BMMSCs on Ti/GO substrate is greatly higher compared to Ti substrate.	[14]
Graphene and GO films on PDMS	PDMS	Human BMMSCs	Osteogenic; Adipogenic	AFM (Atomic Force Microscopy) imaging; Immunofluorescence staining; Alizarin Red staining; Oil Red O staining	Graphene and GO demonstrate to be effective preconcentration platform for growth and differentiation factors. Graphene is useful for osteogenic differentiation, whereas GO strongly enhances adipogenic differentiation.	[13]
GO film	Tissue culture polystyrene	Human ADSCs	Osteogenic; Adipogenic	Cell viability assay; Immunofluorescence staining; Alizarin Red staining; Oil Red O staining	GO film provides a suitable environment for the adhesion, proliferation, and differentiation of human ADSCs. Compared to tissue culture polystyrene, the GO film enhances the differentiation of human ADSCs to osteoblasts and adipocytes, whereas the GO film decreases the chondrogenic differentiation.	[11]
Graphene foam	Tissue culture polystyrene	Human MSCs	Osteogenic	Immunofluorescence staining; SEM (Scanning Electron Microscope) microscopy	Graphene foam allows viability of human MSCs and induces spontaneous osteogenic differentiation.	[22]
Graphene 3D foam	Graphene 2D film	Mouse NSCs	Neuronal	SEM microscopy; Raman spectroscopy; Cell viability assay; Immunofluorescence staining; Western blot; Electrical stimulation	Graphene 3D foam has a greater electrical stimulation performance when compared to graphene 2D film.	[27]
Graphene film on glass	Glass	PC-12 cells	Neuronal	Cell viability assay; ROS assay; LDH (Lactate dehidrogenase) assay; SEM microscopy	Graphene coated glass substrate shows a better PC-12 cells proliferation and neuronal differentiation.	[28]
Graphene coated glass	Glass	Human NSCs	Neuronal	Immunofluorescence staining; Electrical stimulation	Graphene substrate is an excellent cell-adhesion layer during the differentiation process and induces the differentiation of human NSCs more toward neurons than glial cells.	[19]
Graphene film	Tissue culture polystyrene	Mouse NSCs	Neuronal	AFM imaging; Immunofluorescence staining; Electrical stimulation	NSCs seeded on graphene film differentiate and form functional neuronal networks.	[33]
Fluorinated graphene	Graphene	Human BMMSCs	Neuronal	Immunofluorescence staining; AFM imaging;	Fluorinated graphene enhances cell adhesion and proliferation of human BMMSCs. It exhibits a neuro-inductive effect via spontaneous cell polarization.	[29]
rGO/TiO_2_	GO/TiO_2_ and TiO_2_	Human NSCs	Neuronal	flash photo stimulation; Immunofluorescence staining	After flash photo stimulation, human NSCs proliferate more on rGO/TiO_2_ that on GO/TiO_2_ and TiO_2._ The neuronal differentiation of human NSCs on rGO/TiO_2_ substrate is greatly higher compared to GO/TiO_2_ and TiO_2_ substrate.	[34]
GO	Graphene, CNTs	Mouse ESCs	Neuronal	Immunofluorescence staining; Real time PCR	GO substrate demonstrates an important enhancement of dopamine neurons differentiation whereas the GR and the CNTs do not show any important promotion on dopamine neurons differentiation.	[32]

## Graphene and tissue engineering

### Osteogenic differentiation

Bone tissue engineering promises to restore bone defects that are caused by severe trauma, congenital malformations, etc. Many researchers are studying the ways to confer a pro-osteodifferentiation or osteoinductive capability on implants or scaffold materials, where osteogenesis of seed cells is promoted. Graphene provides a new kind of coating material that may confer the pro-osteodifferentiation capability on implants and scaffold materials by surface modification. Here, we review recent studies on the effects of graphene on surface modifications of implants or scaffold materials. The ability of graphene to improve the biological properties of scaffold materials, and its ability to promote the adhesion, proliferation, and osteogenic differentiation of MSCs or osteoblasts have been demonstrated in several studies [20].

**Figure 2:**
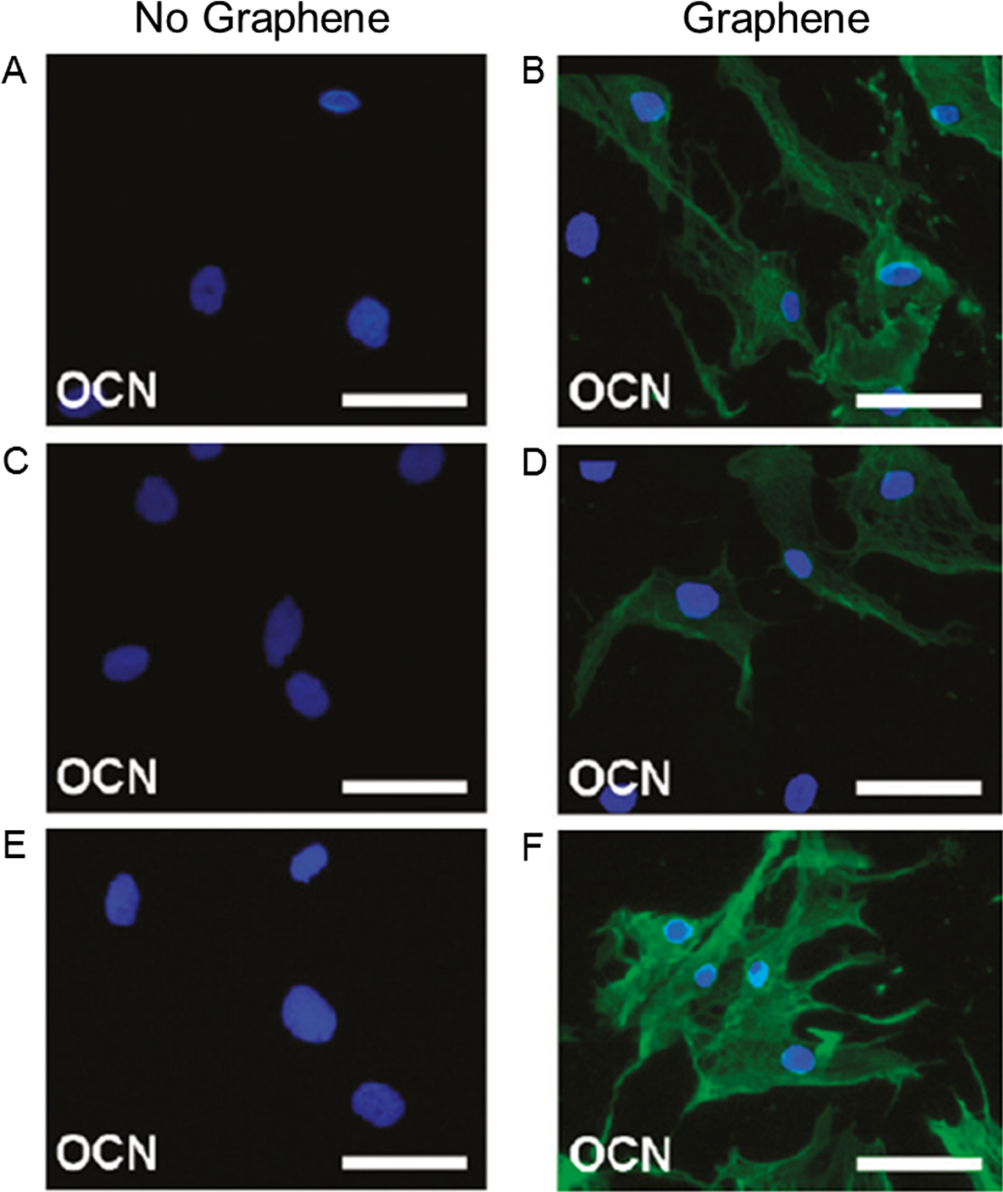
**Immunostaining of MSCs seeded for 15 days in osteogenic differentiation medium on different substrates.** Si/SiO2 **(A,B)**, PDMS **(C,D)**, and PET **(E,F)** substrates were coated or not with graphene. Cells are stained with DAPI (blue) and OCN (green). MSCs growing on Si/SiO2 **(A)**, PDMS **(C)**, and PET **(E)** without graphene show OCN negative staining. Once these substrates are coated with graphene (**B**,**D**,**F** respectively), cells are positive for OCN, indicating osteogenic differentiation. Scale bars are 100 μm [3].

Graphene as a coating material for biocompatible surfaces has proved to be a positive and safe model to obtain osteoblasts starting from MSCs and pre-osteoblasts [17]. Kalbacova et al. made the first observations plating osteoblasts on two different substrates, silica (SiO_2_) and graphene-coated SiO_2_ [21]. After 48 hours incubation the cells were homogenously covering the graphene substrate, while appearing as separate spots on the SiO_2_ surface. The results were supported by fluorescent imaging, revealing that in the graphene substrate the initial number of osteoblasts almost duplicated, compared to a 1,5 increase factor in the SiO_2_ film. Nonetheless, these initial observations tended to be controversial, considering the study of Nayak et al. [3]. When graphene was used as a coating agent on polydimethylsiloxane (PDMS), polyethilentereftalate (PET), glass and Si/SiO_2_, it did not demonstrate to influence the shape or structure of seeded cells compared to uncoated surfaces. Furthermore, these authors investigated the osteogenic potential of graphene on MSCs. MSCs cultured on uncoated Si/SiO_2_, PDMS and PET surfaces showed a CD-44 positive staining and a complete negative osteocalcin (OCN) staining. On the other hand, OCN was significantly represented when cells were cultured on the same graphene-coated surfaces (Figure 2). The results were confirmed by the detection of a greater extent of calcium deposition in the second cells population, confirming the role played by graphene on the induction of osteogenic differentiation.

**Figure 3:**
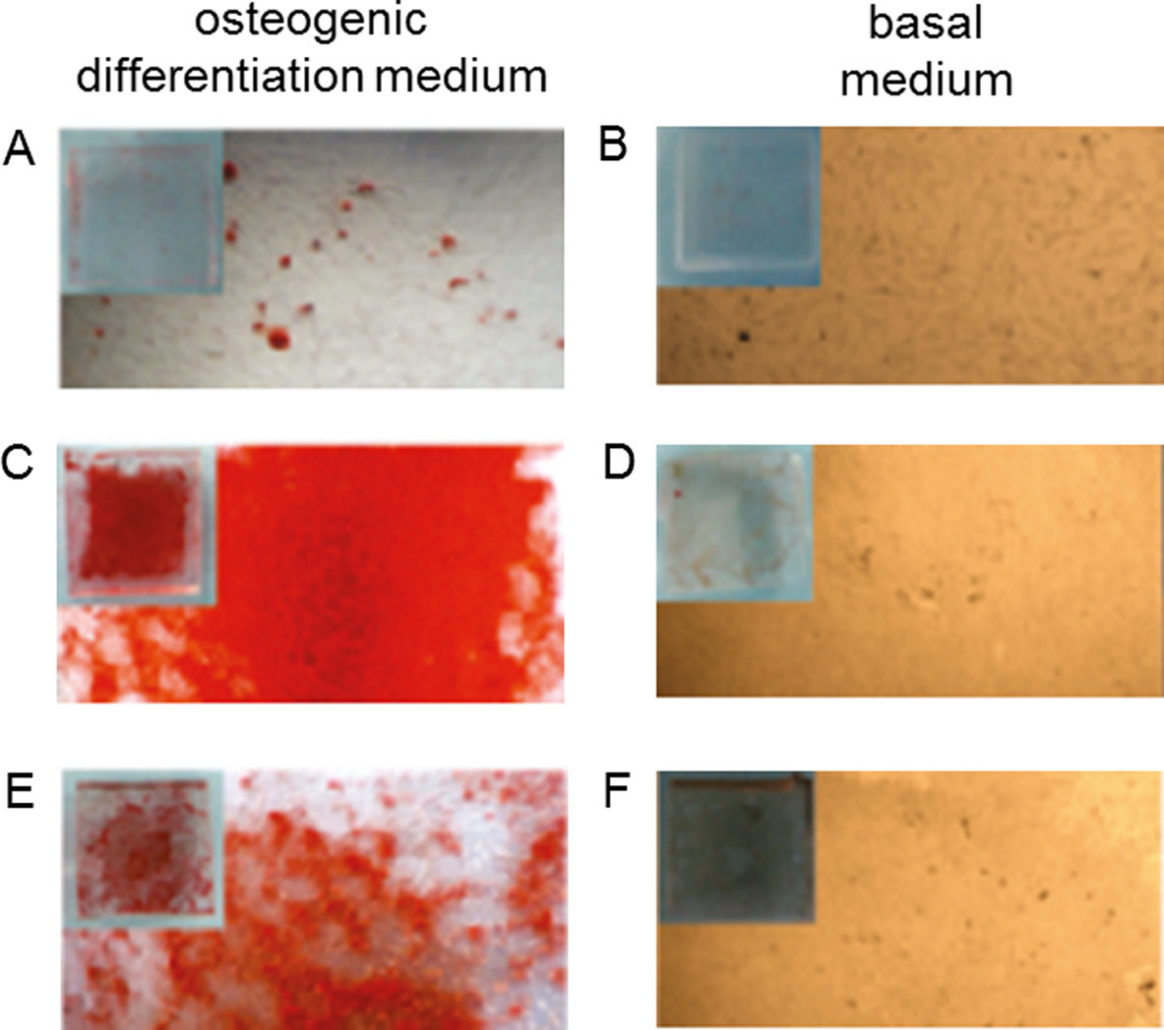
**Alizarin Red staining of MSCs seeded on PDMS, graphene, and GO substrates.** A higher amount of Alizarin Red, which is an indicator of osteogenic differentiation, is found in MSCs cultured on graphene **(C)** for 12 days in osteogenic differentiation medium than that cultured on GO **(E)** and PDMS **(A)** in the same conditions. In presence of basal medium, MSCs do not show staining for Alizarin Red in none of the 3 substrates **(B,** **D,** **F)** [13].

Further studies as the one by Lee et al. showed a positive correlation between culture on graphene substrate and osteogenic differentiation [13]. In particular, the study revealed the ability of graphene substrates to act as a preconcentration platform for osteogenic differentiation factors, such as dexamethasone and beta-glicerophosphate. The osteogenic differentiation was also visualized by Alizarin Red staining (Figure 3).

More recently, the alternative coating with GO has started to be explored. The study by La et al. investigated the potential of GO-coated titanium (Ti) in loading and releasing bone morphogenic protein type 2 (BMP-2) [14]. The osteogenic differentiation of human bone marrow MSCs (hBMMSCs) cultured on Ti and Ti/GO substrates was then tested and immunocytochemical staining for OCN showed a higher osteogenetic potential at 2 and 3 weeks of hBMMSCs on Ti/GO substrate compared to Ti substrate without coating. The same study performed an in vivo validation through implantation of Ti and Ti/GO substrates, with or without BMP-2, into mice calvaria defects. After a 8 weeks follow up, micro computed tomography imaging and the histological analysis confirmed that no new bone formation was observed without the use of BMP-2, but the Ti/GO/BMP-2 implant substrates showed a greater bone formation compared to Ti/BMP-2 substrates.

The osteogenic differentiation potential of MSCs on graphene substrates was confirmed also by the study of Crowder et al., where a 3D graphene structure was used to induce osteogenic differentiation [22]. The authors remarked that the foam shape structure used was particularly suitable as a substrate to induce MSCs to differentiate into bone lineage.

The effect of graphene on MSC osteogenic commitment has been also studied by Duan et al. [23] who compared carbon nanomaterials (CNMs), such as CNT, with graphene. Their combinations with nanofibrous polymeric scaffolds, which mimic the morphology of natural ECM of bone, arouse indeed keen interest in bone tissue engineering. The hypothesis of the authors was that the sheet-like graphene might have stronger enhancement in regulating osteocompatibility than tubular multiwall CNT composite scaffolds, because the former provided more contacting surface to cells than the latter when they were at the same content. Therefore, composite nanofibrous scaffolds were prepared by using poly-L-lactide (PLLA) and graphene as starting materials. Briefly, graphenes were added into PLLA-tetrahydrofuran (THF) solutions, and thermal-induced phase separation (TIPS) technique was applied to induce the nanofibrosis of PLLA. To this end, CNMs were incorporated into nanofibrous PLLA scaffolds by TIPS technique. The CNMs-containing composite nanofibrous scaffolds were biologically evaluated by both *in vitro* co-culture of hBMMSCs and *in vivo* implantation. The nanofibrous structure itself demonstrated significant enhancement in cell adhesion, proliferation and osteogenic differentiation of hBMMSCs, and with the incorporation of CNMs, the composite nanofibrous scaffolds further promoted osteogenic differentiation of hBMMSCs significantly. Between the two CNMs, graphene showed stronger effect in promoting osteogenic differentiation of hBMMSCs than CNT. The results of *in vivo* experiments revealed that the composite nanofibrous scaffolds had both good biocompatibility and strong ability in inducing osteogenesis. CNMs could remarkably enhance the expression of osteogenesis-related proteins as well as the formation of type I collagen. Similarly, the graphene-containing composite nanofibrous scaffolds demonstrated the strongest effect on inducing osteogenesis *in vivo*. These findings demonstrated that CNMs-containing composite nanofibrous scaffolds were obviously more efficient in promoting osteogenesis than pure polymeric scaffolds [23].

The same positive results have been observed by Tavarty et al. [24] that hypothesized that incorporating GO with an osteoinductive material could synergistically direct the differentiation of human MSCs toward osteogenic lineage. Calcium phosphates (CaP) such as hydroxyapatite (HAp) are biomimetic biomaterials that are well-recognized for their osteoconductivity (facilitating bone formation) and osteoinductivity (facilitating the osteogenic differentiation of human MSCs). To validate the above hypothesis, the authors synthesized a novel biocompatible GO-CaP nanocomposite and evaluated its capability of inducing the osteogenic differentiation in human MSCs. The GO-CaP nanocomposite was fabricated using GO microflakes, uniquely structured highly osteoinductive CaP nanoparticles, and pluronics polymeric coating. The osteoinductive properties of GO microflakes, CaP, and GO-CaP on human MSCs were evaluated by quantitative measurements on bone nodule formation and the immunofluorescence imaging of osteoblast biomarkers. GO-CaP exhibited osteogenic capability that was superior to individual or combined effects of GO and CaP. To evaluate the materials’ osteoinductive capability, GO, CaP and GO-CaP were introduced to human MSCs and their osteogenic commitment has been evaluated. Results revealed that treatments with GO, CaP and GO-CaP in osteogenic medium induced significantly larger quantity of calcium than control at all the time points, while no calcification was observed in negative controls. GO-CaP nanocomposites exhibited superior osteoinductivity to CaP or GO, inducing much larger amount of mineralization than control. Phosphate assay was also performed from the deposition in parallel plates after 2 and 3 weeks of treatment. The amount of phosphate among all the groups followed the sequence of GO-CaP > CaP > > GO > control, consistent with the outcome from calcium quantification. Surprisingly, GO microflakes at low concentration (0,5 mg/mL) increased calcification up to 50% more than the control at 3 and 4 weeks. The osteogenic differentiation of human MSCs was verified moreover through immunofluorescence staining of osteoblast markers: alkaline phosphatase (ALP) and OCN after 2 weeks of treatments. In good agreements with Alizarin Red staining, ALP activities and OCN expression level followed the sequence of GO-CaP > CaP > > GO > control, affirming the potential of GO-CaP in directing human MSCs differentiation toward osteogenic lineage [24].

Owing to the superior mechanical properties and low coefficient of thermal expansion, graphene has been widely used in the reinforcement of ceramics. Xie et al. [25] studied that various ratios of graphene (0,5 wt%, 1,5 wt% and 4 wt%) reinforced with graphene calcium silicate (CS) for load-bearing implant surface modification. Surface characteristics of the graphene-calcium silicate (GC) composite coatings were characterized by scanning electron microscopy. Results showed that the graphene plates (less than 4 wt% in the coatings) were embedded in the CS matrix homogeneously. The surfaces of the coatings showed a hierarchical hybrid nano-microstructure, which is believed to be beneficial to the behaviors of the cell and early bone fixation of the implants. Wear resistance measured by a pin-on-disc model exhibited an obvious enhancement with the adoption of graphene plates. The weight losses of the GC coatings decreased with the increase of graphene content. However, too high graphene content (4 wt% or more) made the composite coatings porous and the wear resistance decreased dramatically. The weight loss was only 1,3 ± 0,2 mg for the GC coating containing 1,5 wt% graphene (denoted as GC1,5) with a load of 10 N and sliding distance of 500 m, while that of the pure CS coating reached up to 28,6 ± 0,5 mg. *In vitro* cytocompatibility of the GC1,5 coating was evaluated using a hBMMSCs culture system. The proliferation and ALP, osteopontin and OCN osteogenesis-related gene expression of the cells on the GC1,5 coating did not deteriorate with the adoption of graphene. Conversely, even better adhesion of the hBMMSCs was observed on the GC1,5 coating than on the pure CS coating. All of the results indicate that the GC1,5 coating is a good candidate for load-bearing implants [25].

### Neuronal differentiation

Inducing human NSCs to differentiate into neurons is a critical challenge to reach an important biomedical goal, since a promising opportunity in therapies for neural regeneration could arise [26,27].

Park et al. tested the graphene substrate as a promoter of human NSCs differentiation into neurons [19]. The most remarkable data were observed when comparing the human NSCs seeded on a graphene and a glass substrate after 30 days. Immunofluorescence showed a greater degree of cell attachment along with cell differentiation rate into neurons on the graphene substrate, whereas more glial cells than neurons were found on the glass surface. The analysis was performed counting the immunopositive cells for GFAP (a glial cell marker) and TUJ1 (a neuronal cell marker).

Other results indicating that graphene-based substrates can promote neural differentiation came from the study by Hong et al. [28]. After culturing PC-12 cells on uncovered or graphene-covered glass substrates, the authors observed a better cellular adherence along with higher cell proliferation and neural differentiation on the graphene-coated substrate.

In the attempt of influencing in a significant way stem cell differentiation, several declination of graphene scaffolds were studied. Wang et al. tested MSCs on fluorinated sheets of graphene, observing a strong enhancement of neuronal differentiation when compared to cells seeded on graphene [29]. This indicated how fluorinated graphene may be a good engineered platform to enhance neuronal differentiation. Li et al. used a new 3D scaffold based on graphene, a graphene foam, which regulated mice NSCs behavior supporting their growth and keeping cells at an active proliferation state [27]. Its porous structure showed to be a good substrate for NSCs adhesion, probably due to its irregular surface which improved mechanical adhesion [30]. The scaffold was also proved to be highly biocompatible since no cytotoxicity was observed and cell viability was not affected. Moreover, the 3D structure had a greater electrical stimulation performance when compared to a 2D graphene structure, and electrical stimulation has been proven to induce neural differentiation [31].

The study by Yang et al. investigated the capability of GO, graphene and CNTs to induces dopamine neural differentiation of mouse embryonic stem cells (ESCs) [32]. ESCs were seeded in all the 3 substrates and a stromal cell-derived inducing activity method was used. After 14 days of differentiation, the GO substrate demonstrated an important enhance of dopamine neural differentiation while the graphene and CNTs did not show any important promotion of dopamine neural differentiation.

**Figure 4:**
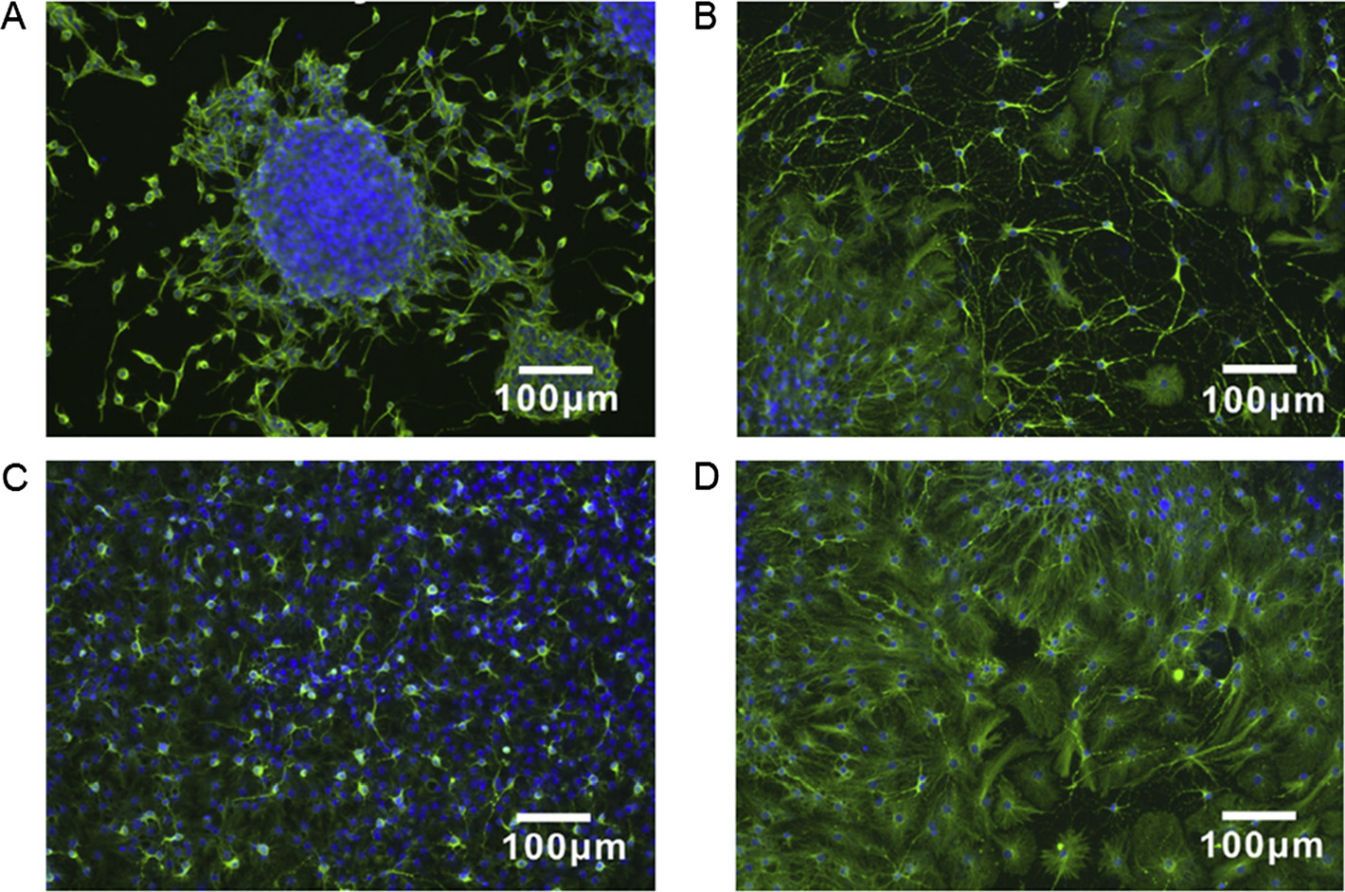
**Immunostaining of neurospheres deriving from NSCs seeded on graphene films up to14 days.** Cells are stained with DAPI (blue) and beta-tubulin (green) at day 1 **(A)**, day 3 **(B)**, day 7 **(C)**, and day 14 **(D)**. NSCs differentiated on graphene substrates display neurites growing to various directions and distances, indicating the development of neural networks [29].

Another important characteristic of graphene could be represented by its capability to form a functional neural network. In the study by Tang et al., neurospheres were seeded on graphene substrates and after a 14 days culture the process of network formation was made clear by beta-tubulin immunostaining [33]. Newly formed neuritis started to form synapses. This result confirmed how graphene could be considered as a valid substrate to promote neural activity (Figure 4).

Stimulating human NSCs to differentiate into neurons rather than glia is a another key point in order to obtain a neural regeneration result [29]. The study by Akhavan and Ghaderi specifically concentrated on testing human NSCs differentiating behavior on different substrates (TiO_2_, GO/TiO_2_, and rGO/TiO_2_), with and without flash photo stimulation [34]. When the 3 substrate went under flash photo stimulation, the number of cells increased by a factor of 1,5 on the rGO/TiO_2_ substrate, by a rate of 48% in the GO/TiO_2_ substrate and by a rate of 24% on the TiO_2_ substrate. No differences were detected without flash stimulation. When the differentiation rate was analyzed it was found that the rGO/TiO_2_ substrate was the more beneficiary of the flash photo stimulation: an 88% decrease in the ratio of glial cells and an 81% increase in the ratio of neuronal cells. In the other 2 substrates the difference was less evident: 25% of neuronal cells increase rate on the GO/TiO_2_ substrate and 15% on the TiO_2_ substrate. Overall, flash photo stimulation of graphene-based substrate provide both a greater cell proliferation and a greater human NSCs differentiation into neurons rather that glia cells. However, these results could be obtained when the flash photo stimulation of human NSCs was at an optimum concentration of a biocompatible hole scavenger, and at an optimum flash intensity.

For central nervous system (CNS) regeneration, the selective differentiation of NSCs into either neurons or oligodendrocytes (as opposed to astrocytes) is highly desirable. Several approaches have been employed to guide differentiation into neurons, however, oligodendrocyte differentiation has proven to be much more elusive, resulting in only a small percentage of the differentiated cell population. The primary approach to guide oligodendrocyte differentiation has focused on either developing culture media containing a combination of growth factors or the forced expression of key oligodendrocyte-promoting transcription factors. In this view the developing a biomaterials-approach to achieve efficient differentiation of NSCs into mature oligodendrocytes, while eliminating the potential adverse or variable side-effects from growth factors and viral gene vectors, would be highly beneficial.

Shah et al. [35] developed a graphene-based nanomaterial for the design of hybrid nanofibrous scaffolds to guide NSCs differentiation into oligodendrocytes. The authors demonstrated as the use of GO was an effective coating material in combination with electrospun nanofibers for the selective differentiation of NSCs into oligodendrocytes. By varying the amount of GO coating on the nanofibers, they observed a GO concentration-dependent change in the expression of key neural markers, wherein coating with a higher concentration of GO was seen to promote differentiation into mature oligodendrocytes. Further investigation into the role of GO-coating on the nanofibrous scaffolds showed the overexpression of a number of key integrin-related intracellular signaling molecules that are known to promote oligodendrocyte differentiation in normal development. In their studies, polycaprolactone (PCL) was electrospun onto a metallic collector and then transferred to glass substrates for cell culture using a medical grade adhesive. Nanofibers with an average diameter of 200–300 nm were generated, which is a fiber size range that has been reported to be favorable for oligodendrocyte culture, potentially due to the close morphological resemblance to axons. Thin-layered GO was then synthesized and then deposited on the PCL nanofiber surface. Among the various cell-signaling proteins, the authors examined the expression of focal adhesion kinase (FAK), Akt, integrin-linked kinase (ILK) and Fyn kinase (Fyn), which have been found to mediate cytoskeletal remodeling and process extension during oligodendrocyte development. The researchers found that NSCs cultured on the GO coated surfaces enhanced the gene expression of all of these factors. These signaling molecules exhibited the same trend in expression, wherein the GO-coated glass showed higher expression than PCL, and PCL-GO showed the strongest level of expression with a 2,6-fold increase in FAK and about a 1,7-fold increase in Akt, ILK and Fyn. Additionally, treating the cells grown on PCL-GO scaffolds with cell signaling inhibitors showed a significant decrease in gene expression of mature oligodendrocyte markers, which provides further evidence for the potential role of such cellular signaling in the observed oligodendrocyte differentiation. Collectively, this data supports the role of GO-coating in the upregulation of these downstream molecules in the integrin signaling pathway and may explain, at least in part, the enhanced oligodendrocyte differentiation of NSCs on hybrid scaffolds. Data obtained suggest that the GO-coating on the nanofiber scaffolds may promote oligodendrocyte differentiation through specific microenvironmental interactions which activate integrin-related intracellular signaling. Overall, Shah et al. demonstrated the capability of a unique graphene-nanofiber hybrid scaffold to provide instructive physical cues that lead to the selective differentiation of NSCs into mature oligodendrocytes, without introducing differentiation inducers in the culture media. The ability to selectively guide stem cell differentiation by merely changing the properties of an underlying biomaterial scaffold is a valuable approach for tissue engineering, which can help complement or potentially eliminate the use of exogenous differentiation. Moreover, their hybrid scaffold is exceptional in that it combines the well-established properties of nanofibers and graphene-based nanomaterials. For instance, nanofibers have been shown to provide ideal topography for fabricating nerve guidance conduits, directing neurite outgrowth and promoting axonal regeneration. On the other hand, graphene-based nanomaterials provide permissive surfaces for protein and cell adhesion, as well as high conductivity to mediate electrical stimulation for supporting neuronal electrophysiology [35].

GO based nanoparticles has been used also to drive the commitment of ESCs into dopamine neurons. Yang et al. [36] studied the effects of CNTs, GO and graphene nanoparticles on the dopamine neural differentiation of mouse ESCs. The dopamine neural differentiation of the ESCs was examined by immunocytochemistry and real-time PCR showing that only GO could effectively promote dopamine neuron differentiation after induction of a stromal cell-derived inducing activity and further enhance dopamine neuron-related gene expression compared with cells treated with no nanoparticle control, and the other two nanoparticles (CNTs and graphene). In conclusion, authors suggest that GO is a promising nanomaterial-based technical platform to effectively enhance dopamine neural differentiation of ESCs, which can be potentially applied for cell transplantation therapy.

Novel important applications of graphene in neuroscience has been found in the end by Song at al. [37], who studied the anti-inflammatory effects of three-dimensional graphene foams cultured with microglial cells. Nanomaterials are increasingly used in medical diagnosis and treatment due to their unique mechanical, optical, electrical, and magnetic properties. However, studies have revealed that most of the nanomaterials could initiate some form of inflammation both *in vitro* and *in vivo*, then lead to other biological effects, as expected of any foreign particulate. Current studies mostly focus on the pulmonary inflammation caused by nanomaterials insults, while little is known about the neuroinflammatory effects. Given that some nanomaterials (quantum dots, CNTs, graphene, etc.) have been attempted to be used in neuroscience, the neuroinflammation should be considered. External insults range from hypoxia and ischemia to a number of bacterial and viralinfections, all of which elicit a characteristic neuroinflammatory reaction in the brain. Microglia, astrocytes, and peripheral macrophages are key players mediating this response. In the brain, most of the damage caused by nanomaterials is mediated by the microglia, a macrophage-like, phagocytic cell that is normally inactive unless confronted by potentially damaging xenobiotics. In response to certain cues such as brain injury or immunological stimuli, however, microglia are readily activated. Graphene has been at the forefront of nanotechnology and advanced materials sciences due to its intriguing physical and chemical features. Especially, it has been utilized in a variety of biomedical applications. Recently, Li et al. [38] discovered the great potentials of using graphene for neural interfacing, as it could promote neurite sprouting and outgrowth in primary culture of hippocampal neurons, enhance the neural performances in the network differentiated by NSCs, direct stem cell differentiation, and be used as electric field stimulator for effective cerebral blood volume enhancement. Meanwhile, graphene foams were found to greatly induce NSCs differentiation to neuronal lineage and were proposed as a neural scaffold for NSC-based therapy. These pioneering works demonstrate the capability of graphene for applications in CNS. However, to the best of our knowledge, there is no report regarding the possible neuroinflammatory effects of graphene, which should be well addressed before any further clinical applications. In this work, the researchers report the neuroinflammatory responses of microglia under the presence of graphene by *in vitro* culturing and justify whether this graphene-induced neuroinflammatory effects are detrimental or beneficial to the neural cells. The importance of this work is the elucidation of the pro- and/or anti-inflammatory effects of graphene and pave the way for the applications of graphene in biomedicine. The graphene, especially 3D graphene, supported microglia growth and showed comparable biocompatibility to the commercial tissue culture polystyrene substrates. Despite of the similar proinflammatory responses in the microglia without lipopolysaccharide (LPS) activation, 3D graphene evoked much milder neuroinflammation in the microglia after LPS activation in comparison to 2D graphene, suggesting that the topographical structures of the materials might affect the inflammatory behaviors. Furthermore, the unique topographical structures of 3D-graphene may restrict the morphological transformation of microglia under over-activation, leading to the anti-inflammatory effects.

### Adipogenic differentiation

Graphene and GO substrates have been also used for investigating their effects on the adipogenic differentiation of MSCs. In particular, in the study of Lee et al., MSCs were plated on graphene or GO sheets in presence of adipogenic differentiation medium for 14 days [13]. The cells were then stained with Oil Red O and counted. The results showed a strong suppression of adipogenesis on graphene substrates; on the contrary, GO was a strong enhancer. This difference was probably due to the ability of graphene to denaturate insulin: GO did not denaturate insulin which therefore could maintain its role of mediator of fatty acid synthesis.

Kim et al. evaluated GO potential in differentiating human adipose-derived stem cells (ADSCs) into adipocytes, demonstrating a higher adipogenesis on the GO substrate when compared to the control (tissue culture polystyrene) [11]. This study also documented how GO could be considered a unique substrate, allowing the attachment and proliferation of ADSCs and modulating cell differentiation not only toward the adipogenic line but also toward the osteogenic and epithelial phenotypes. On the contrary, the GO films resulted in decreased chondrogenic differentiation of the ADSCs.

### Periodontal ligament stem cells

In regenerative dentistry, stem cell-based therapy often requires a scaffold to deliver cells or growth factors to the injured site. GO and silk fibroin (SF) are promising biomaterials for tissue engineering as they are both non toxic and promote cell proliferation. A field that could be improved by the availability of an effective film scaffold would be the reparation of periodontal tissues. The periodontium, which is composed of four dental tissues (i.e., gingiva, alveolar bone, cementum, and periodontal ligament [PDL]), is constantly maintained by periodontal ligament stem cells (PDLSCs) owing to their great capacity of differentiation into cementoblasts, odontoblasts, and fibroblasts. PDL plays a key role in the attachment of teeth to the jaw; in the most drastic cases of periodontitis, which is associated with a chronic inflammation process, PDL destruction could lead to the loss of the tooth [39]. With the aim of expanding tissue engineering therapy to as many patients as possible, acellular biomaterials may be employed as a novel approach to heal periodontal site by the active recruitment of autologous cells into the PDL scaffold, thus providing an in situ regeneration in cases of periodontitis. So, in order to make the evaluation of the mentioned SF-GO composite film as scaffold, human dental stem cells were chosen by Rodriguez-Lonzano et al. as cellular model to test the the performance of GO and SF. In their study the authors evaluated the effects of the novel biomaterials GO and SF on PDLSCs phenotype, adhesion, proliferation rate and viability. Biocompatibility of scaffolds is a prerequisite for generating cell-biomaterial constructs and for their successful clinical application. GO and fibroin-based biomaterials have been previously studied for several tissue engineering based-therapies [39], but they have never been tested in conjunction with mesenchymal stem cells isolated from PDL. The morphology of PDLSCs cultured for different times on GO and fibroin coated surfaces by staining of actin cytoskeleton showed that PDLSCs cultured on fibroin displayed lower amounts of F-actin, lower spreading and delayed growth. By contrast, GO or GO plus fibroin-coated surfaces significantly improved F-actin content, cell spreading and growth rate from 96 h of culture when compared to fibroin alone. Furthermore, it has been also studied the proliferation rate of PDLSCs on GO and fibroin-based biomaterials by MTT assays. Results confirmed that after 7 days of culture, PDLSCs showed a high cell proliferation rate in presence of GO, although it was slightly lower than on plastic, whereas fibroin or GO-fibroin biomaterials supported a discrete proliferation. In addition, to evaluate the possible cellular cytotoxic effect of the different biomaterials employed as well as changes on the expression of mesenchymal surface markers, the researchers characterized their surface molecule expression pattern by flow cytometry. Culture of PDLSCs on fibroin, GO or GO plus fibroin did not significantly alter the level of expression of CD73,CD90 or CD105 after 24, 48, 72, 96 or 168 h compared to expression levels displayed by PDLSCs cultured on plastic. Thus, the biomaterials employed in this study were able to maintain the mesenchymal phenotype of PDLSCs.

### Cardiomyogenic differentiation

As well reported above, graphene has drawn attention as a substrate for stem cell culture and has been reported to stimulate the differentiation of multipotent adult stem cells. Recently, Lee et al. [40] reported that graphene enhances the cardiomyogenic differentiation of human ESCs at least in part, due to nanoroughness of graphene coated with vitronectin (VN). Human ESCs were cultured on either VN-coated glass or VN-coated graphene for 21 days. The cells were also cultured on glass coated with Matrigel, which is a substrate used in conventional, directed cardiomyogenic differentiation systems. Results confirmed that the culture of human ESCs on graphene promoted the expression of genes involved in the stepwise differentiation into mesodermal and endodermal lineage cells and subsequently cardiomyogenic differentiation compared with the culture on glass or Matrigel. In addition, the culture on graphene enhanced the gene expression of cardiac-specific extracellular matrices. The authors concluded in the end that graphene may provide a new platform for the development of stem cell therapies for ischemic heart diseases by enhancing the cardiomyogenic differentiation of human ESCs.

The same results have been reched by Park et al. [41] that demonstrated the use of MSCs culture to promote cardiomyogenic differentiation. Also in this case graphene exhibited no sign of cytotoxicity for stem cell culture. MSCs were committed toward cardiomyogenic lineage by simply culturing them on graphene. The authors speculated that this may be attributed, at least partially, to the regulation of expression levels of ECM and signaling molecules.

### iPSCs

The successful reprogramming of somatic cells into induced pluripotent stem cells (iPSCs) by Takahashi and Yamanaka in 2006 [42] was seen as a landmark event in disease research. This new technology promised to provide a powerful tool for modeling human pathology that could be used to understand the underlying causes of various human diseases. The following years saw a stream of new and improved approaches for converting somatic cells into more differentiated cell types.

In an interesting study by Yoo et al. [43], the authors reported that graphene promotes the reprogramming of mouse somatic fibroblasts into iPSCs.

The generation of ESCs-like cells from somatic cells by ectopic expression of defined factors is an approach commonly known as cell reprogramming. Moreover, because iPSCs generation is known to be a multiple-step process mediated by overexpression of the transcription factors Oct4, Sox2, Klf4, and cMyc, the generation of iPSCs is very inefficient, and cell reprogramming is considered a stochastic process in which successive barriers must be overcome to reach a state of pluripotency. In particular, one of the first noticeable changes during the reprogramming of somatic fibroblasts is their transformation into tightly packed clusters of rounded cells in a process that resembles a mesenchymal-to-epithelial-transition (MET). More interestingly, recent studies found that microtopography substrate affects the MET, improving reprogramming efficiency. The authors examinated whether somatic fibroblasts should be efficiently reprogrammed into the pluripotent state on graphene-based substrate. Fitstly they carachterised the monolayer graphene film by Raman spectroscopy, then they studied whether the graphene substrate enhances cell reprogramming by seeding Oct4-GFP knock-in (KI) mouse embryonic fibroblasts (MEFs) onto both the control and graphene-coated substrate in MEF medium. One day after plating, MEFs were transduced using specific vectors expressing Oct4, Sox2, Klf4, and cMyc transcription factor. The MEFs plated on the graphene substrate began to form colonies 10 days after viral infection, exibiting a significant increase in colonies undergoing reprogramming on graphene substrate. To quantify the reprogramming efficiency, they performed FACS analysis for Oct4-GFP-positive iPSCs derived from Oct4-GFP KI MEFs in both the control substrate and graphene-coated substrate. Fifteen days after doxycycline (dox) induction, the graphene substrate cultures had a significant increase in the number of GFP-cells and quantitative real-time PCR analysis showed that pluripotency marker genes, including Oct4, Nanog, Sox2 and Esrrb were markedly elevated in graphene-coated substrate cultures compared to uncoated substrate cultures. The pluripotent state of graphene-induced iPSCs was assessed by immunostaining of pluripotency marker. Consistent with these results, they observed that the graphene-coated substrate significantly increased the number of Oct4, SSEA1 and Nanog colonies. Moreover, in order to analyze the differentiation potential of graphene mediated iPSCs, their capacity for direct differentiation into the cell types of the three germ layers has been tested. Immunocytochemical staining and real-time PCR showed that the differentiated cells were positive. In the end the further developmental potency of the graphene induced iPSC lines was investigated by SCID mice. Four weeks after injection, teratomas were readily visible and histological analysis confirmed the presence of cell types derived from all three embryonic germ layers validating the pluripotency of these graphene induced iPS cells. In order to examine whether graphene substrate affects chromatin state during reprogramming, they measured the enrichment level of histone modifications that mark active (histone 3 lysine 4 tri-methylation, H3K4me3) in the graphene induced cell reprogramming. Surprisingly, they found a dramatic increase of H3K4me3 expression on graphene substrate and that the transcription start sites of Oct4 and Nanog were enriched for H3K4me3 in graphene surface-induced reprogramming cultures. Taken together, these results suggest that the graphene-coated substrate specifically promotes the MET process in cell reprogramming without affecting the EMT process. In this study, the autors showed that the epigenetic reprogramming of somatic fibroblasts is enhanced on graphene substrate providing a potential method for the efficient generation of iPSCs. Interestingly, they found a specific induction of MET by the graphene substrate during reprogramming and an accumulation of H3K4me3, which facilitates increased Oct4 and Nanog occupancy. These data indicate a unique role for graphene substrates in facilitating cell reprogramming [43].

Carbon nanoparticles such as zero dimensional (0D) fullerenes, one dimensional (1D) CNTs, and recently two dimensional (2D) graphene have been investigated for applications in therapeutics, bioimaging, and regenerative medicine. MSCs are currently being widely investigated to repair, regenerate, and restore damaged tissues. Nanoparticles have been employed to deliver growth factors or genes into MSCs to manipulate their differentiation [44]. The development of graphene nanoparticles for MSC applications necessitates thorough examination of their effects and interactions with these cells to identify potential therapeutic doses. To date, very few studies have investigated the cytotoxicity of graphene nanoparticle formulations with specific focus on progenitor cells or MSCs. Zhang et al. examined the toxicity of graphene quantum dots, single reduced graphene sheets with diameters in the range of 5–10 nm, on three progenitor cell types: neurospheres cells, pancreas progenitor cells, and cardiac progenitor cells [45]. Akhavan et al. employed umbilical cord-derived MSCs and investigated the size-dependent cytotoxicity of graphene oxide nanoplatelets and reduced graphene oxide nanoplatelets (prepared using the modified Hummer’s method) [46]. Graphene nanoparticles, depending on the synthesis method, can exhibit different morphologies, chemical properties, and physical properties. Thus, it is necessary to systematically investigate the effects that graphene nanoparticles with different morphologies, synthesized by various methods, have on MSC viability and differentiation. In an interesting study of Taluktat et al. [44], the dose- and time-dependent effects were investigated of three graphene nanoparticles on the viability and differentiation of human MSCs. The initial cytotoxicity screening over a broad range of concentrations (0–300 μg/mL) and time points (one and three days) was performed on ADSCs and BMMSCs to identify a range of potentially safe doses. ADSCs were then employed to investigate whether these graphene nanoparticles at a potentially safe low and high dose affect the differential capabilities of MSCs. The cytotoxicity and differentiation studies together allowed identification of the range of doses for the three-graphene nanoparticle formulations that do not elicit any significantly adverse outcomes on the viability and differentiation capabilities of MSCs. Results confirmed that graphene nano-onions (GNOs), graphene oxide nanoribbons (GONRs), and graphene oxide nanoplatelets (GONPs) elicited a dose-dependent (0–300 μg/mL), but not a time-dependent (24 and 72 h) cytotoxic response on ADSCs and BMMSCs. For all three nanoparticles, concentrations of less than 50 mg/mL showed no significant differences compared to untreated controls. The adipogenic and osteogenic differentiation potential of ADSCs was not adversely affected after treatment with a low (10 mg/mL) or high (50 mg/mL) concentration. GNOs and GONPs were internalized by ADSCs, while GONRs were not. The results suggest that GNOs, GONRs, and GONPs at concentrations of less than 50 mg/mL for 24 or 72 h could be considered potentially safe incubation conditions for ex vivo labeling for MSCs. The results open avenues for use of these graphene nanoparticle formulations for ex vivo labeling of MSCs for applications in regenerative medicine.

## Conclusions

The literature on the biological interactions of graphene family is growing rapidly, and includes studies primarily motivated by biomedical applications, and environmental health and safety. As with other biomaterials, the issue of potential toxicity arises not only in biomedical applications, but also in non-biomedical products where unintended occupational, consumer, and environmental exposures can occur. The exact mechanism of GO and the derived composites in SCs differentiation is still unresolved. It has been generally hypothesized that the surface characteristics of graphene family nanomaterials such as nanotopography, surface stiffness, and large absorption capacity influence the molecular pathways that control the fate of stem cells. Both G and GO were reported acting as preconcentrators for chemicals, proteins and growth factors on their surface to promote cell differentiation.

In this view, we have reported a review of the international literature to produce a state-of-the-art update of graphene applied to tissue engineering and stem cells.

Many studies confirmed that graphene and its related materials are able to induce human stem cells differentiation into specific lineages. Materials coated with graphene or GO or even 3D graphene foam were capable to guarantee viability and to induce osteogenic differentiation of stem cells when compared with traditional substrates or scaffolds [3,11,13,14,21,22]. In terms of neuronal regeneration, graphene and GO showed the same promising differentiative potential. Furthermore, a real cellular and electrical network could be structurally and functionally created on these materials [19,27–29,32–34]. As well as in osteogenic and neuronal differentiation, GO can control adipogenesis [11,13].

Even if we are still uncertain about potential in vivo applications, mostly due to open questions around toxicity, graphene clearly appears to be a step forward in the field of tissue engineering.

In this light it is interesting the results found by Chen et al. that express an expert opinion based on scientometric patterns revealed by CiteSpace without prior working experience in the regenerative medicine field. Emerging trends and new developments have been identified based on structural and temporal properties derived from the relevant publications. The detected surge of the keyword graphene in the literature of regenerative medicine led the authors to investigate the nature and context of its use in regenerative medicine. The investigation revealed a rapidly increasing number of studies that specifically used graphene and GO in regenerative medicine research because of their desirable surface properties for, among other applications, culturing and maintaining SCs. Similarly a detected burst of citations provides insightful guidance for navigating through the fast-changing [47].

There is also an emerging literature on potential health risks. Despite the popular image of graphene as a large-area substrate coating, many graphene-family materials are dry powders at some point in their processing, and in this form pose the most significant exposure risk through inhalation. Of particular concern are FLG samples directly following the thermal exfoliation step or after washing and drying. There is need for measurement of airborne dust levels in research laboratories, and in pilot and full-scale manufacturing facilities. GFNs are high-surface-area materials with corresponding high potential to cause adsorptive and quenching artifacts in biological assays [48]. Adsorption on carbon surfaces is generally favored for molecules with low solubility, partial hydrophobicity, or positive charge (for the common case of negatively charged GFNs). The biological consequences may include (i) micronutrient depletion, (ii) artifacts in assays that rely on dye-based molecular probes (iii) the capacity to carry small molecule drug cargoes, and (iv) synergistic or antagonistic toxic effects when GFNs coexist with small molecule toxicants, whose bioavailabilty can be increased or decreased as they partition to graphene surfaces. More work is needed in this area, and authors must be skeptical of standard assays without extensive controls for possible interference. As a final note,in the area of toxicity, there have been a number of studies reported, but the field is too young and the literature too limited to reach conclusions about potential hazards sufficient for risk assessment or regulation. Nano-GO has been reported to be biocompatible in a number of the studies focused on biomedical applications, at least under the limited conditions covered by such studies. Other studies have reported adverse biological responses, including cytotoxicity using human lung epithelial cells and fibroblasts [49,50]. Cellular uptake of GFNs has been shown for macrophages and human lung epithelial cells in some studies although there have been no studies exploring the mechanism of uptake and intracellular fate. These sheet-like GFNs may physically perturb cytoskeletal organization, mitosis, organelle integrity, and impair cell motility and secretion. A potential toxicity pathway for GFNs is oxidative stress [51,52] it is not clear whether oxidant generation is related to reactive edge sites or an indirect response of target cells to nanomaterials. It is clear that lateral size is a key variable in cell uptake, while layer number affects deposition and surface area, and surface chemistry has a large affect on adsorption and dispersibility. Molecular dyanamics modeling of interactions between GFNs and cell membranes should provide valuable information about uptake mechanisms. Systematic investigation of toxicological endpoints using a defined set of carbon nanomaterials including carbon black, CNT, and GFNs will be important to develop structure-activity relations. Because graphenes form a material family with wide variation in properties, the graphene-bio field will benefit greatly in the long run, if its authors show diligence in characterizing their materials, and describing them according to layer number, lateral size, surface chemistry rather than *ad hoc* sample names.

## Abbreviations

ADSCs: Adipose-derived stem cells
BMP-2: Bone morphogenic protein type 2
CaP: Calcium phosphates
CNMs: Carbon nanomaterials
CNS: Central nervous system
CNT: Carbon nanotube
CS: Calcium silicate
dox: Doxycycline
ECM: Extracellular matrix
ESCs: Embryonic stem cells
FAK: Focal adhesion kinase
FLG: Few-layer-graphene
Fyn: Fyn kinase
GC: graphene-calcium silicate
GFNs: Graphene-family nanomaterials”
GNOs: Graphene nano-onions
GNS: Graphene nanosheets
GO: Graphene oxide
GONPs: Graphene oxide nanoplatelets
GONRs: Graphene oxide nanoribbons
H3K4me3: Histone 3 lysine 4 tri-methylation
HAp: Hydroxyapatite
hBMMSCs: Human bone marrow MSCs
ILK: Integrin-linked kinase
iPSCs: Induced pluripotent stem cells
KI: Knock-in
LPS: Lipopolysaccharide
MET: Mesenchymal-to-epithelial-transition
MSCs: Mesenchymal stem cells
NSCs: Neural stem cells
OCN: Osteocalcin
PCL: Polycaprolactone
PDL: Periodontal ligament
PDLSCs: Periodontal ligament stem cells
PDMS: Polydimethylsiloxane
PET: Polyethilentereftalate
PLLA: Poly-L-lactide
rGO: Reduced graphene oxide
SF: Silk fibroin
SiO2: Silica
THF: Tetrahydrofuran
Ti: Titanium
TIPS: Thermal-induced phase separation
VN: Vitronectin
